# Evaluation of the cytotoxic effect and antibacterial, antifungal, and antiviral activities of *Hypericum triquetrifolium* Turra essential oils from Tunisia

**DOI:** 10.1186/1472-6882-13-24

**Published:** 2013-01-29

**Authors:** Zyed Rouis, Nabil Abid, Sadok Koudja, Thabet Yangui, Ameur Elaissi, Pier Luigi Cioni, Guido Flamini, Mahjoub Aouni

**Affiliations:** 1 Laboratoire des Maladies Transmissibles et Substances Biologiquement Actives LR99ES27, Faculty of Pharmacy, University of Monastir, Monastir, Tunisia; 2 Laboratoire d’Analyse, Traitement et Valorisation des Polluants de l’Environnement et des Produits, Faculté de Pharmacie Rue Avicenne, Monastir 5000, Tunisia; 3 Laboratoire des Bioprocédés, Pôle d’Excellence Régionale AUF, (PER-LBP) Centre de Biotechnologie de Sfax, BP: 1177, Sfax 3018, Tunisia; 4 Laboratory of Pharmacognosy, Faculty of Pharmacy, University of Monastir, Monastir, Tunisia; 5 Dipartimento di Chimica Bioorganica e Biofarmacia, Universita’ di Pisa, Via Bonanno 33, Pisa 56126, Italy

**Keywords:** *Hypericum triquetrifolium*, Coxsakievirus B3, Essential oils, Bacteria, Fungi

## Abstract

**Background:**

A number of bio-active secondary metabolites have been identified and reported for several *Hypericum* species. Many studies have reported the potential use of the plant extracts against several pathogens. However, *Hypericum triquetrifolium* is one of the least studied species for its antimicrobial activity. The aim of the present study was to evaluate the cytotoxic effect of the essential oils of *Hypericum triquetrifolium* as well as their antimicrobial potential against coxsakievirus B3 and a range of bacterial and fungal strains.

**Methods:**

The essential oils of *Hypericum triquetrifolium* harvested from five different Tunisian localities (Fondouk DJedid, Bou Arada, Bahra, Fernana and Dhrea Ben Jouder) were evaluated for their antimicrobial activities by micro-broth dilution methods against bacterial and fungal strains. In addition, the cytotoxic effect and the antiviral activity of these oils were carried out using Vero cell lines and coxsakievirus B3.

**Results:**

The results showed a good antibacterial activities against a wide range of bacterial strains, MIC values ranging between 0.39-12.50 mg/ml and MBC values between 1.56-25.0 mg/ml. In addition, the essential oils showed promising antifungal activity with MIC values ranging between 0.39 μg/mL and 12.50 μg/mL; MFC values ranged between 3.12 μg/mL and 25.00 μg/mL; a significant anticandidal activity was noted (MIC values comprised between 0.39 μg/mL and 12.50 μg/mL). Although their low cytotoxic effect (CC_50_ ranged between 0.58 mg/mL and 12.00 mg/mL), the essential oils did not show antiviral activity against coxsakievirus B3.

**Conclusion:**

The essential oils obtained from *Hypericum triquetrifolium* can be used as antimicrobial agents and could be safe at non cytotoxic doses. As shown for the tested essential oils, comparative analysis need to be undertaken to better characterize also the antimicrobial activities of *Hypericum triquetrifolium* extracts with different solvents as well as their purified fractions and their pure secondary metabolites.

## Background

Essential oils are aromatic extracts which have been used since ancient times as flavouring agents and constituents of several commercial products. The chemical composition of essential oils is often variable among different plants and even between different plant parts. In addition, the composition may also differ according to the site of collection (geographical provenance), as their components play a major role in the plant adaptation to the ecology and the environment, including biotic and abiotic factors [1,2]. Currently, the use of essential oils is more common today than ever before due to their increasing demand for food, cosmetics and pharmaceutical industries. In addition, the interest in essential oils has increased as potential alternatives for therapeutic purposes against common microbes. Bacterial resistance is spreading throughout the world primarily due to the excessive use of antibiotics and poor infection control practices in hospitals, making it one of our times biggest issues [3]. Scientific literature revealed the antimicrobial, antifungal and antioxidant potentials of several essential oils [4,5]. In addition, the antiviral potential of essential oils has been well documented [6,7].

Microorganisms such as *Staphylococcus aureus* (*S. aureus*), *Staphylococcus epidermidis* (*S. epidermidis*), *Enterococcus faecalis* (*E. faecalis*), *Pseudomonas aeruginosa* (*P. aeruginosa*), and *Escherichia coli* (*E. coli*) are frequently isolated from skin wounds in humans and animals. In addition, *S. epidermidis* infections are commonly acquired in hospitals as a result of contamination of surgical cuts with microorganisms from the patients themselves or from the hospital personnel [8]. Infection with *P. aeruginosa* is one of the most serious complication in burn patients [9,10], followed by infections with *E. coli*, *S. aureus* and other microorganisms [9]. Infection with *Bacillus cereus* has been well documented in the literature for over a century and it is generally associated with gastroenteritis caused by the consumption of infected food. *Vibrio alginolyticus* is ubiquitous in seawater and tends to cause superficial wound and ear infections (otitis media and otitis externa) [11]; this infection can progress to bacteraemia and necrotising fasciitis, particularly in the immunocompromised patients [12]. *Vibrio cholerae* (*V. cholerae*), a Gram (−) bacterium and the causative agent of cholera, has caused several pandemics since 1816, as well as sporadic inter-epidemic outbreaks. *V. cholerae* is autochthonous in a region of the world where cholera never occurs and that the human body is not an obligate environment for the presence and dispersal of this organism [13]. *Salmonella typhimurium* causes typhoid fever associated with gastroenteritis; the infection is caused by consuming contaminated food or drinks. *Aeromonas hydrophila* (*A. hydrophila*) has been receiving increasing attention both as an opportunistic and as a primary pathogen of humans, aquatic and terrestrial animals [14]. *A. hydrophila* inhabits aquatic environments and the gastrointestinal tract of healthy fish. It also commonly occurs in foods, milk, red meats and poultry [15-17]. It causes disease and mortality mainly in freshwater fish but sometimes in marine fish [17]. The bacterium also infects humans and causes lesions ranging from gastroenteritis to septicaemia [18].

The genus *Hypericum* is a member of the *Hypericaceae* family [19,20]. A number of bio-active secondary metabolites have been identified and reported for several *Hypericum* species [21-23]. Essential oils extracted from *Hypericum* species are well documented for their antimicrobial activities [4,24-33].

*Hypericum triquetrifolium* Turra (*H. triquetrifolium*), native to Eastern Europe and the Mediterranean area, has been traditionally used for its sedative, antihelminthic, anti-inflammatory, and antiseptic effects [24,34]. In addition, several studies have reported the potential use of its essential oil and crude extracts as therapeutic substances, mainly in the treatment of burns, gastroenteritis, antinociceptive and antioxidant drugs [35-37]. However, *H. triquetrifolium* is one of the least studied species for its antimicrobial activity. According to literature data, only a previous study using the growth inhibition assay for a number of bacterial and candidal strains [38] is reported for *H. triquetrifolium*.

In the present study, the antimicrobial, cytotoxic effect and the antiviral activities of the essential oils extracted from *H. triquetrifolium* from five different Tunisian localities were evaluated *in vitro*. The variation in their activities was discussed according to their chemical compositions previously reported [39].

## Methods

### Plant material and essential oil extraction

Voucher specimens identified by Prof. Mohammed El Hedi El Ouni (Department of Biology, Faculty of Sciences of Bizerte, Tunisia) have been deposited in the Herbarium of the Laboratory of Transmissible Diseases and Biological Active Substances (Faculty of Pharmacy of Monastir, Tunisia), under the following accession codes: *H. tri.* 1, *H. tri.* 2, *H. tri.* 3, *H. tri.* 4, and *H. tri.* 5 for *Hypericum triquetrifolium* from Bou Arada, Bahra, Dhrea Ben Jouder, Fernana and Fondouk Djedid, respectively.

Aerial parts (the top 25 cm) of the plant have been collected during full blooming from five different Tunisian localities between June and July 2008. In brief, plant samples were air-dried in darkness at room temperature for one week. Then, samples (500 g) were cut in small pieces and subjected to hydro-distillation for 3 h, using the standard apparatus recommended by the European Pharmacopoeia. The obtained oils were stored at +4°C in glass vials until analysis. The resultant oils were studied for their chemical variability using Gas Chromatography – Electron Ionization Mass Spectrometry (GC-EIMS) and GC coupled with Chemical Ionization Mass Spectrometry (GC/CIMS). The results are reported in a previous work [39].

### Cells and tested microorganisms

#### Cell line

The Vero cells were derived from the kidney of a normal, adult, African green monkey (Cercopithecus) in 1962, by Yasumura and Kawakita at the Chiba University in Japan. This cell line has been extensively used for virus replication studies and plaque assays. Vero cells (kindly provided by Pr. Bruno Pozzetto, Laboratory of Bacteriology-Virology, Saint-Etienne, France) were used for culturing enterovirus strains. Vero cells were maintained in RPMI 1640 supplemented with 10% fetal bovine serum (FBS), L-Glutamin (2 mM), penicillin (100 U/mL), and streptomycin (100 *μ*g/mL). Cells were incubated at 37°C in a 5% CO_2_ humidified atmosphere.

### Bacterial and fungal strains

Gram (+) and Gram (−) bacterial strains were used in the present study (Table 1). In addition, fungal and yeast strains and isolates were included for the analysis of the fungicidal activity of the essential oils (Table 2).

**Table 1:** Bacterial reference strains and their pathological effects

**Bacterial strains**	**Catalogue number**	**Effects**
*Bacillus cereus*^*a*^	ATCC 11778	Foodborn
*Escherichia coli*^*b*^	ATCC 35218	Foodborn
*Vibrio alginolyticus*^*b*^	ATCC 17749	Intestinal diseases, wound and ear infections
*Vibrio cholerae*^*b*^	ATCC 39315	Cholera
*Pseudomonas aeruginosa*^*b*^	ATCC 27853	Gastrointestinal diseases
*Salmonella typhimurium*^*b*^	CIP 104115	Typhoid fever
*Aeromonas hydrophila*^*b*^	ATCC 7966	Gastroenteritis and Cellulitis
*Enterococcus faecalis*^*a*^	ATCC 29212	Endocardites
*Staphylococcus aureus*^*a*^	ATCC 25923	Foodborn, scalded skin syndrome
*Staphylococcus epidermidis*^*a*^	CIP 106510	Nosocomial

^*a*^Gram (+) bacteria.

^*b*^Gram (−) bacteria.

**Table 2:** Fungal and candidal strains and their effects

**Fungal and yeast strains**	**Catalogue number/ isolates**	**Effects**
*Aspergillus niger*	CTM 10099	Black mold on certain fruits and vegetables, contaminant of food, aspergillosis, otomycosis, damage to the ear canal and tympanic membrane.
*Fusarium solani*	Isolated from Tomato plants	Damping off on certain fruits and vegetables, keratitis, endophthalmitis, cutaneous infections, burn patients, mycetoma, onychomycosis, sinusitis, pulmonary disease, endocarditis, catheter infections, and septic arthritis
*Botrytis cinerea*	Isolated from strawberry fruit	Winegrower’s lung, Hypersensitivity pneumonitis, Grey mould affects many plant species
*Candida albicans*	ATCC 90028	Candidiasis, opportunistic oral and genital infections
*Candida glabrata*	ATCC 90030	Pathogen for the urogenital tract, and for the bloodstream (fungemia*)*
*Candida krusei*	ATCC 6258	Fungemia, nosocomial pathogen

### Virus strain

Coxsakievirus B3 Nancy strain (kindly provided by Pr. Bruno Pozzetto, Laboratory of Bacteriology-Virology, Saint-Etienne, France) was propagated in Vero cells. In brief, 100 *μ*L of the virus suspension were used to infect a confluent monolayer of Vero cells in 75 cm^2^ culture flask and adsorbed for 1 h to allow viruses to adhere onto the cells. Non-adherent particles were washed off using 2% RPMI 1640 medium and the infected cells overlaid with 20 mL of 2% RPMI 1640 and incubated again until full cytopathic effect was observed in five to six days. The harvested virus was stored at −70°C until used.

### Antimicrobial activities

#### Minimum inhibitory and minimum bactericidal concentrations (MIC and MBC)

The minimum inhibitory concentration (MIC) values for each essential oil against the tested bacterial strains and environmental isolates were determined according to the standard protocols [36]. The bacterial strains were cultured in tryptic soy broth (TSB) or agar (Sigma, Tunis, Tunisia) at the appropriat temperature for the strain (30°C or 37°C). Inocula were prepared by adjusting the turbidity of each bacterial culture to reach an optical density of 0.5 McFarland standards, corresponding to approximately 1 – 5 × 10^8^ CFU/mL. The concentration of spore suspensions was determined using a haematocytometer (Thoma cell) and adjusted to 1 – 5 × 10^7^ spores/mL. The broth dilution method was carried out in 96-well microtitre plates using microbial reference strains and field isolates. The essential oils were prepared aseptically and transferred to sterile 96-well microtitre plates by two-fold serial dilutions using 5% dimethylsulfoxide (DMSO) and then diluted in TSB. The resultant doses of the tested essential oils ranged between two and 250 *μ*g/mL. Eighty microliters of the prepared oil suspension were added to each well, followed by 10 *μ*L of each oil dose and 10 *μ*L of resazurin indicator solution (7-Hydroxy-3H-phenoxazin-3-one 10-oxide). The latter reagent allows the detection of microbial growth in extremely small volumes of solution in microtitre plates without using a spectrophotometer. Two control wells were used for each plate: one well containing microorganism and resazurin and a second well containing only medium and resazurin (in order to check the sterile conditions of the experiment). The plates were incubated anaerobically at 37°C for 24 h. After incubation, bacterial growth was evaluated by color change from blue to pink. The lowest dose indicating inhibition of growth was recorded as the MIC.

To determine the MBC, 10 *μ*L of each culture medium with no visible growth were removed from each well and inoculated in TSB plates. The CFU values of surviving organisms were determined after aerobic incubation at the appropriated temperature during 16 – 20 hours [40].

### Minimium inhibitory and minimium fungicidal concentrations (MIC and MFC)

The fungicidal activity was evaluated as discussed above. The only differences consisted of the culture of fungi and the yeast strains on malt extract broth (MEB) or agar (Fluka, Madrid, Spain) and incubation at 28°C. The essential oils (diluted in 5% DMSO) at different doses were mixed with MEB and the plates were incubated anaerobically at 25°C for 48 hours.

### Cytotoxicity assay

The evaluation of the cytotoxic effect of the essential oils is based on the reduction of MTT (3-[4,5-dimethylthiazol-2-yl]-2,5-diphenyl tetrazolium bromide), by the mitochondrial dehydrogenase of viable cells, to give a blue formazan product that can be measured spectrophotometrically [41]. Cells were seeded in 96-well plates at a concentration of 5 × 10^4^ cells per well and incubated at 37°C for 24 h in a 5% CO_2_ humidified atmosphere. After treatment with various doses of the essential oils (0.19, 0.39, 0.78, 1.56, 3.12, 6.25, 12.50, and 25.00 mg/mL), the cells were incubated at 37°C for an additional 48 hours. The cells were examined daily under a phase-contrast microscope to determine the minimum dose of the tested essential oil that induced alterations in cell morphology. At this stage, the medium was removed and cells in each well were incubated for 3–4 hours at 37°C with 100 *μ*L of MTT solution (5 mg/mL). MTT solution was then discarded and 50 *μ*L DMSO were added to dissolve insoluble formazan crystals. Optical density (OD) was measured at 540 nm using a standard microplate reader (BIO-TEK® EL×800™ Universal Microplate Reader, NY, USA). Cell viability was expressed with respect to the absorbance of the control wells (untreated cells), which were considered 100% of absorbance. The percentage of cytotoxicity was calculated as [(A-B)/A] × 100; where A and B are the OD_540_ of untreated and treated cells, respectively. The 50% cytotoxic concentration (CC_50_) was defined as the dose of the essential oil required for the reduction of cell viability by 50%, which were calculated by regression analysis.

### Virus inhibition assay

In this assay, essential oils were tested for their possible use either to cure infected cells or to protect them from infection. The experiment is simple, and relies on a cell culture system able to support virus growth. Confluent Vero cells were treated with the essential oils at three different doses (CC_50_, ½ CC_50_, ¼ CC_50_) during and after virus infection in two sets of experiments as follows: (Experiment 1) 5 × 10^4^ TCID_50_ of the virus were exposed to three doses (CC_50_, 1/2 CC_50_, 1/4 CC_50_) of each essential oil for one hour at 37°C. Then 100 *μ*L of the mixture were added to the cells cultured fluently in 96-well flat-bottom microtiter plate; (Experiment 2) Cells were treated with three doses (CC_50_, 1/2 CC_50_, 1/4 CC_50_) of each essential oil (100 *μ*L) for one hour at 37°C. Then, 5 × 10^4^ TCID_50_ of the virus (100 *μ*L) were added.

Altogether, the experiment aims to test the mode of action of the essential oils and to evaluate any effect of the essential oils on the virus (Experiment 1) or on the cells before the infection (Experiment 2).

All the plates were incubated in a CO_2_-incubator for 48 hrs. The viability of the infected and non-infected cells was evaluated using MTT reduction assay, as described above. The percent of protection was calculated as follows:

$$\begin{array}{l} \mathrm{Percentprotection}=\left[\left(\mathrm{ODT}\right)V-\left(\mathrm{ODC}\right)V\right]\\ \;/\left[\left(\mathrm{ODC}\right)M-\left(\mathrm{ODC}\right)V\right]\times 100 \end{array}$$

Where (ODT) V, (ODC) V and (ODC) M indicate the absorbance of the sample, the virus-infected control (no compound) and mock-infected control (no virus and no compound), respectively [42].

### Statistical analysis

Data of antibacterial and antifungal activities were subjected to statistiical analysis using Principal Components (PCA) and Hierarchical Clusters Analysis (HCA). Statistical tests were performed using STATISTICA-Pc Software 9.0 (Stat Soft Inc, http://www.statsoft.com).

## Results

### Antibacterial activity

As shown in Table 3, the essential oils exhibited different antimicrobial activities with respect to the geographical region of origin.

**Table 3:** Antibacterial activity of the essential oils of H. triquetrifolium (MIC / MBC; mg/mL)

**Bacterial strains**	H. tri. B.A.*	H. tri. Bah.*	H. tri. D.B.J.*	H. tri. Fer.*	H. tri. F.Dj.*
*Bacillus cereus* ATCC 11778^*a*^	25.00/25.00	12.50/12.50	25.00/25.00	6.25/12.50	1.56/3.12
*Enterococcus feacalis* ATCC 29212^*a*^	12.50/12.50	0.39/0.39	12.50/12.50	6.25/12.50	0.39/3.12
*Staphylococcus aureus* ATCC 25923^*a*^	3.12/3.12	25.00/25.00	12.50/25.00	6.25/25.00	0.78/3.12
*Staphylococcus epidermidis* CIP 106510^*a*^	12.50/25.00	25.00/25.00	12.50/25.00	25.00/25.00	1.56/3.12
*Vibrio alginoliticus* ATCC 17749^*b*^	12.50/25.00	12.50/12.50	12.50/12.50	6.25/12.50	1.56/3.12
*Escherichia coli* ATCC 35218^*b*^	12.50/25.00	25.00/25.00	25.00/25.00	6.25/12.50	1.56/6.25
*Vibrio cholerae* ATCC 39315^*b*^	0.39/25.00	12.50/25.00	0.78/3.12	3.12/3.12	25.00/25.00
*Pseudomonas aeruginosa* ATCC 27853^*b*^	6.25/6.25	12.50/12.50	25.00/25.00	6.25/6.25	0.39/1.56
*Salmonella typhimirium* CIP 104^*b*^	0.39/12.50	6.25/25.00	25.00/25.00	6.25/25.00	0.78/6.25
*Aeromonas hydrophila* ATCC 7966^*b*^	0.39/6.25	0.39/3.12	12.50/25.00	6.25/12.50	3.12/6.25

^*a*^Gram (+) bacteria, ^*b*^Gram (−) bacteria, MIC: Minimium Inhibitory Concentration; MBC: Minimium Fungicidal Concentration. H. tri. B.A.: *Hypericum triquetrifolium* collected from Bou Arada; H. tri. Bah.: *Hypericum triquetrifolium* collected from Bahra; H. tri. D.B.J.: *Hypericum triquetrifolium* collected from Dhrea Ben Jouder; H. tri. Fer.: *Hypericum triquetrifolium* collected from Fernana; H. tri. F.Dj.: *Hypericum triquetrifolium* collected from Fondouk Djedid.

*SD values = 0.00.

The essential oil of *H. triquetrifolium* from Fondouk DJedid (F.DJ.) showed a more potent antibacterial activity against the tested strains (MIC range: 0.39 – 1.56 mg/mL; MBC range: 1.56 – 6.25 mg/mL), with the exception of *Vibrio cholerae* (MIC = MBC = 25 mg/mL).

The essential oil of *H. triquetrifolium* collected in Bou Arada (B.A.) was comparatively more bacteriostatic against Gram (−) bacterial strains (MIC range: 0.39 – 12.50 mg/mL; MBC range: 6.25 – 25.00 mg/mL). However, the highest bactericidal effect was detected against *S. aureus* (MIC = MBC = 3.12 mg/mL).

The essential oils obtained from western regions, Bahra (Bah.) and Fernana (Fer.), were lesser active. Essential oils from Bahra showed a good activity against *E. feacalis* and *A. hydrophila* (MIC = 0.39 mg/mL for both strains; MBC range: 0.39 – 3.12 mg/mL, respectively).

The essential oils from Dhrea Ben Jouder (D.B.J.) exhibited a weaker antimicrobial activity against the tested strains (MIC range: 12.50 – 25.00 mg/mL; MBC range: 12.50 – 25.00 mg/mL). On the contrary, a significant activity (MIC = 0.78 mg/mL; MBC = 3.12 mg/mL) was detected against *Vibrio cholerae*.

Altogether, with the exception of the essential oils from F.DJ., all the remaining samples showed a bacteriostatic effect against *V. cholerae,* with MIC values ranging between 0.39 mg/mL and 12.50 mg/mL. MBC values were 3.12 mg/mL and 25.00 mg/mL for the essential oils from Fer./D.B.J. and B.A./Bah., respectively. In addition, *B. cereus*, *S. epidermidis*, *E. coli* and *V. alginoliticus* were resistant to all the essential oils, but sensitive to the essential oils from F.DJ., with MIC and MBC values ranging between 6.25 and 25.00 mg/mL.

**Figure 1:**
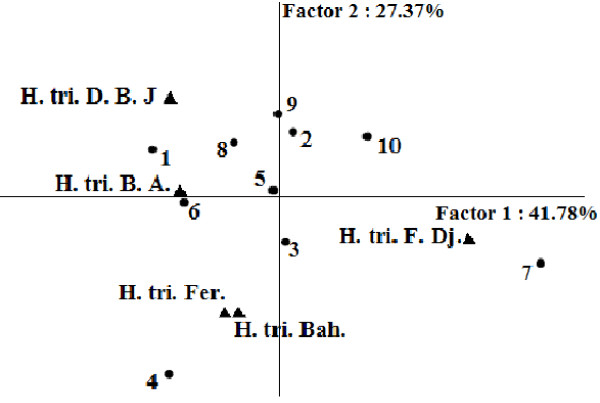
**PCA Projection of the *H. Triquetrifolium *essential oils based upon their bacteriostatic activities against tested strains.** H. tri. B. A. *H. triquetrifolium* harvested in Bou Arada, H. tri. Bah. *H. triquetrifolium* harvested in Bahra, H. tri. D. B. J. *H. triquetrifolium* harvested in Dhrea Ben Jouder, H. tri. Fer. *H. triquetrifolium* harvested in Fernana, H. tri. F. Dj. *H. triquetrifolium* harvested in Fondouk Djedid. 1. *Bacillus cereus,* 2. *Enterococcus feacalis,* 3. *Staphylococcus aureus.* 4. *Staphylococcus epidermidis.* 5. *Vibrio alginoliticus.* 6. *Escherichia coli.* 7. *Vibrio cholera.* 8. *Pseudomonas aeruginosa.* 9. *Salmonella typhimirium.* 10. *Aeromonas hydrophila.*

**Figure 2:**
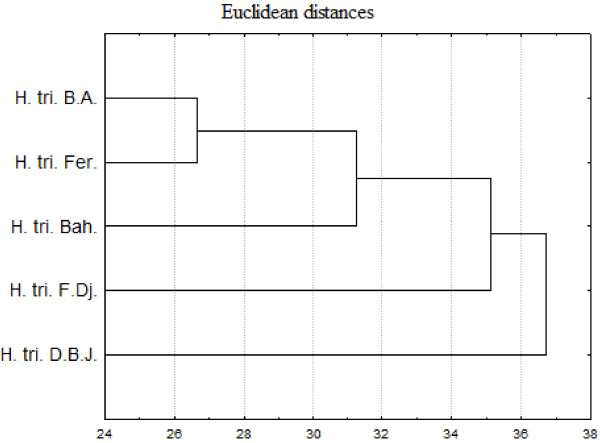
**Dendrogram obtained by hierarchical analysis based on the Euclidean distances between groups of bacteriostatic activities of studied essential oils.** H. tri. B. A. *H. triquetrifolium* harvested in Bou Arada, H. tri. Bah. *H. triquetrifolium* harvested in Bahra, H. tri. D. B. J. *H. triquetrifolium* harvested in Dhrea Ben Jouder, H. tri. Fer. *H. triquetrifolium* harvested in Fernana, H. tri. F. Dj. *H. triquetrifolium* harvested in Fondouk Djedid.

In the PCA analysis of the bacteriostatic results (Figure 1), the horizontal axis explains 41.78% of the total variance, while the vertical axis a further 27.37%. This classification was supported HCA analysis which evidenced three groups (I, II, and III), identified by their MIC with a dissimilarity ≥ 32.0 (Figure 2). When the dissimilarity was ≥ 30.0, group I was divided into two subgroups (I_a_ and I_b_). The horizontal axis permitted the separation of group IV from the other groups whereas the axis II separated group V from all the other groups. Group I was represented by essential oils of *H. triquetrifolium* from Bou Arada, Bahra, and Fernana. These essential oils were characterized by their weak activities against *Bacillus cereus*, *Pseudomonas aeruginosa*, *Escherichia coli*, *Staphylococcus epidermidis* and *Vibrio alginoliticus* and their potent effects against *Aeromonas hydrophila*. Subgroup I_b_, limited to the sole sample from Bahra was distinguished from the other oils of subgroup I_a_ by its good activity against *Enterococcus feacalis* and its weak activity against *Vibrio cholerae*. The variability within subgroup I_a_ was due to the effects against *Staphylococcus aureus* and *Salmonella typhimirium*. These two strains were resistant to the action of the oil from Fernana and sensitive to the one of Bou Arada.

Group II was represented by the oil of *H. triquetrifolum* obtained from plants harvested in Fondouk Djedid. This oil was characterized for its strong activity against all the tested strains, except *Vibrio cholerae* that was sensitive for the majority of the other oils.

Group III was limited to the essential oil of plant harvested in Dhrea Ben Jouder and it is distinguished from all the other groups by its week activity against all the tested strains, with the exception of *Vibrio cholerae*.

**Figure 3:**
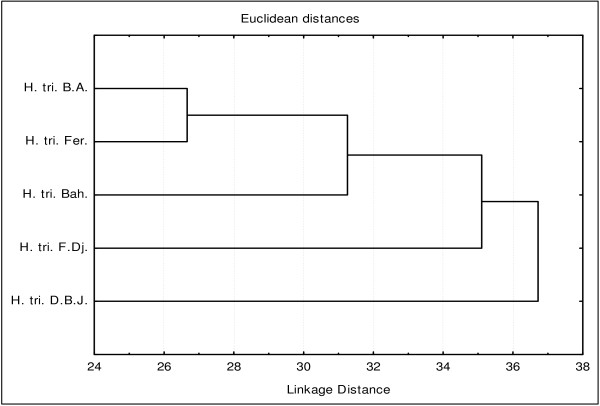
**Dendrogram obtained by hierarchical analysis based on the Euclidean distances between groups of bactericid activities of studied essential oils.** H. tri. B. A. *H. triquetrifolium* harvested in Bou Arada, H. tri. Bah. *H. triquetrifolium* harvested in Bahra, H. tri. D. B. J. *H. triquetrifolium* harvested in Dhrea Ben Jouder, H. tri. Fer. *H. triquetrifolium* harvested in Fernana, H. tri. F. Dj. *H. triquetrifolium* harvested in Fondouk Djedid.

For the bactericidal activity, based on Euclidean coefficient matrix, the HCA classified the studied populations in four groups, with dissimilarity <28: group A (*H. triquetrifolium* from D. B. J.), group B (*H. triquetrifolium* from F. Dj.), group C (*H. triquetrifolium* from Bah.), and group D (*H. triquetrifolium* from Fer. and B. A.), (Figure 3).

**Figure 4:**
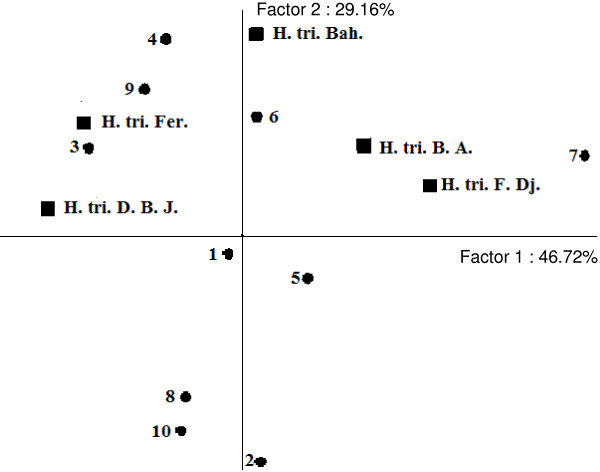
**PCA Projection of the *H. Triquetrifolium *essential oils based upon their bactericid activities against tested strains.** H. tri. B. A. *H. triquetrifolium* harvested in Bou Arada, H. tri. Bah. *H. triquetrifolium* harvested in Bahra, H. tri. D. B. J. *H. triquetrifolium* harvested in Dhrea Ben Jouder, H. tri. Fer. *H. triquetrifolium* harvested in Fernana, H. tri. F. Dj. *H. triquetrifolium* harvested in Fondouk Djedid. 1. *Bacillus cereus,* 2. *Enterococcus feacalis,* 3. *Staphylococcus aureus.* 4. *Staphylococcus epidermidis.* 5. *Vibrio alginoliticus.* 6. *Escherichia coli.* 7. *Vibrio cholera.* 8. *Pseudomonas aeruginosa.* 9. *Salmonella typhimirium.* 10. *Aeromonas hydrophila.*

This classification was supported by PCA that resumed 75.88% of the total variability. In the 2-D plan, the horizontal axis (axis 1) explained 46.72% of the total variance, whereas the vertical axis (axis 2) showed further 29.16% of variance (Figure 4). PCA analysis showed that group A was characterized by its moderate activity against *Vibrio cholerae*. Group B was distinguished by a moderate bactericidal activity against all the tested strains, except *Vibrio cholerae*.

Group C was characterized by its potent activity against *Enterococcus feacalis* which was resistant to the action of the other oils.

Contrary to all other groups, group D was characterized by a moderate bactericidal activity against two or more strains (*Aeromonas hydrophila*, *Pseudomonas aeruginosa*, and *Staphylococcus epidermidis* for Bou Arada samples, and against *Vibrio cholerae* and *Pseudomonas aeruginosa* for Fernana essential oil).

### Antifungal activity

As shown in Table 4, the essential oils of *H. triquetrifolium* exhibited a better antifungal and candidal activities than antibacterial activity, with MIC values ranging between 0.39 *μ*g/mL and 12.50 *μ*g/mL whereas MFC values were within the 1.56 μg/mL and 25.00 μg/mL range.

**Table 4:** Antifungal activity of essential oils of H. triquetrifolium against fungal and yeast strains (MIC / MFC; μg/mL)

**Strains**	H. tri. B.A.*	H. tri. Bah.*	H. tri. D.B.J.*	H. tri. Fer.*	H. tri. F.Dj.*
*Aspergillus niger*	3.12/3.12	12.50/12.50	6.25/6.25	12.50/12.50	3.12/3.12
*Fusarium solani*	3.12/3.12	12.50/12.50	6.25/6.25	3.12/3.12	3.12/3.12
*Botrytis cinerea*	3.12/3.12	12.50/12.50	6.25/6.25	3.12/3.12	3.12/3.12
*Candida krusei* ATCC 6258	0.39/25.00	6.25/6.25	3.12/25.00	3.12/6.25	6.25/6.25
*Candida albicans* ATCC 90028	0.39/25.00	6.25/6.25	3.12/25.00	6.25/6.25	3.12/6.25
*Candida glabrata* ATCC 90030	0.39/1.56	3.12/6.25	1.56/25.00	6.25/6.25	1.56/6.25

MIC: Minimium Inhibitory Concentration; MBC: Minimium Fungicidal Concentration.

H. tri. B.A.: *Hypericum triquetrifolium* collected from Bou Arada; H. tri. Bah.: *Hypericum triquetrifolium* collected from Bahra; H. tri. D.B.J.: *Hypericum triquetrifolium* collected from Dhrea Ben Jouder; H. tri. Fer.: *Hypericum triquetrifolium* collected from Fernana; H. tri. F.Dj.: *Hypericum triquetrifolium* collected from Fondouk Djedid.

*SD values = 0.00.

The essentials oil from B.A. showed the most potent fungistatic activity, with MIC values of 0.39 *μ*g/mL and 3.12 *μ*g/mL for candidal and filamentous strains (*Aspergillus niger*, *Fusarium solani* and *Botrytis cinerea*), respectively. The best antifungal activity was exerted against *C. glabrata* (MFC = 1.56 *μ*g/mL).

The essential oils from the Estern regions of Tunisia (F.DJ. and D.B.J.) showed a more potent antifungal activity against the tested candidal strains, with MIC values ranging between 1.56 *μ*g/mL and 6.25 *μ*g/mL. The essential oils from F.DJ. showed a more potent antifungal activity against filamentous fungal strains (MIC = 3.12 *μ*g/mL, MFC = 3.12 *μ*g/mL) than the essential oils from D.B.J. (MIC = 6.25 *μ*g/mL, MFC = 6.25 *μ*g/mL). In addition, the essential oils from F.DJ. (MIC range: 1.56 – 6.25 *μ*g/mL, MFC = 6.25 *μ*g/mL) showed slightly higher anticandidal activity than the essential oils from D.B.J. (MIC range: 1.56 – 3.12 *μ*g/mL, MFC = 25.00 *μ*g/mL).

Compared to the eastern regions, essential oils from the western localities (Bah. and Fer.) were less fungistatic, with MIC values ranging between 3.12 *μ*g/mL and 12.50 *μ*g/mL, while similar fungicidal activity, with MFC values ranging between 3.12 *μ*g/mL and 12.50 *μ*g/mL.

**Figure 5:**
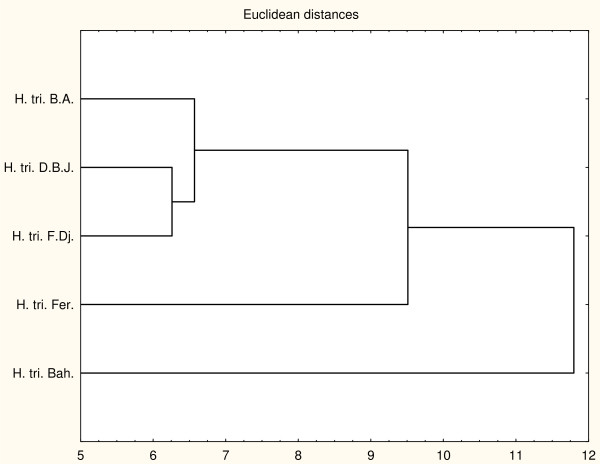
**Dendrogram obtained by hierarchical analysis based on the Euclidean distances between groups of fongistatic activities of studied essential oils.** H. tri. B. A. *H. triquetrifolium* harvested in Bou Arada, H. tri. Bah. *H. triquetrifolium* harvested in Bahra, H. tri. D. B. J. *H. triquetrifolium* harvested in Dhrea Ben Jouder, H. tri. Fer. *H. triquetrifolium* harvested in Fernana, H. tri. F. Dj. *H. triquetrifolium* harvested in Fondouk Djedid.

The HCA analysis for the five essential oils based on their CMI against fungi permitted to evidence three groups (A, B, and C) at a distance of dissimilarity <7 (Figure 5). The first group was limited to the sole sample from Bahra and it was characterized by its poor activity against all mycetes (MIC = 12.50 mg/ml) and its moderate activity on yeast strains. The essential oil obtained from plants harvested in Fernana constituted Group B, which was characterized by a moderate activity against all fungi strains, except *Aspergillus niger* which was more resistant to the action of this oil (MIC = 12.50 mg/ml). The third group was formed by the oil samples from Fondouk Djedid, Dhrea Ben Jouder, and Bou Arada. This group was separated from the others because of their strong to moderate activities against all the tested strains. When the dissimilarity was ≥ 6.25, group III was divided into two subgroups (III_a_, and III_b_). Subgroup III_a_ was formed by Fondouk Djedid and Dhrea Ben Jouder samples and it was characterized by a relatively strong activity against *Candida glabrata.* Subgroup III_b_ was limited to the Bou Arada oil and showed a potent activity against the three tested yeast strains.

**Figure 6:**
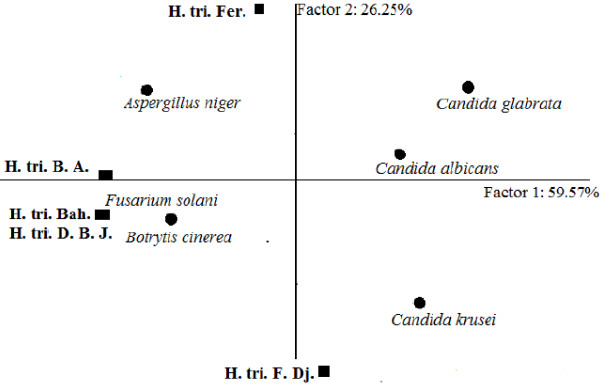
**PCA Projection of the *H. Triquetrifolium *essential oils based upon their fongistatic activities against tested strains.** H. tri. B. A. *H. triquetrifolium* harvested in Bou Arada, H. tri. Bah. *H. triquetrifolium* harvested in Bahra, H. tri. D. B. J. *H. triquetrifolium* harvested in Dhrea Ben Jouder, H. tri. Fer. *H. triquetrifolium* harvested in Fernana, H. tri. F. Dj. *H. triquetrifolium* harvested in Fondouk Djedid. 1. *Aspergillus niger*. 2. *Fusarium solani*. 3. *Botrytis cinerea*. 4. *Candida krusei*. 5. *Candida albicans*. 6. *Candida glabrata*.

PCA analysis performed with MIC values of the five essential oils against the tested fungi explained 85.28% of total variance using the two first factors (Figure 6). In particular, the first axis explained 59.57% of the total variance, while the vertical axis a further 26.25%. According to this analysis, the essential oils of plants harvested from Bou Arada, Bahra, and Dhrea Ben Jouder were negatively correlated with axis 1 and with yeast strains (which were positively correlated with the horizontal factor); this group of oils correlated positively with mycetes strains (negatively correlated with horizontal axis). This observation suggests that these oils were relatively more potent against yeast strains than mycetes strains. The essential oil of plant collected from Fondouk Djedid correlated negatively with the second factor and it was characterized by its weak activity against *Candida krusei*. Sample from Fernana correlated positively with the second axis. It was distinguished by its relatively good activity on *Candida krusei* and its weak action against *Aspergillus niger*.

Statistical analysis based on MFC values against fungi strains showed that while essential oils were classified according to their activities against mycetes and yeast strains respectively using the PCA analysis, they were discriminated according to the total of their activities with the HCA analysis.

**Figure 7:**
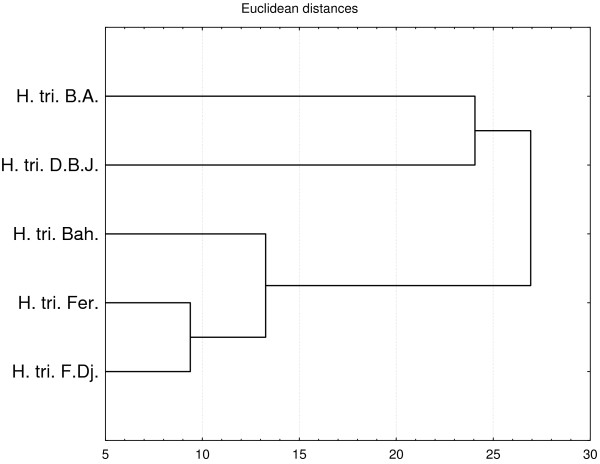
**Dendrogram obtained by hierarchical analysis based on the Euclidean distances between groups of fongicid activities of studied essential oils.** H. tri. B. A. *H. triquetrifolium* harvested in Bou Arada, H. tri. Bah. *H. triquetrifolium* harvested in Bahra, H. tri. D. B. J. *H. triquetrifolium* harvested in Dhrea Ben Jouder, H. tri. Fer. *H. triquetrifolium* harvested in Fernana, H. tri. F. Dj. *H. triquetrifolium* harvested in Fondouk Djedid.

HCA analysis classified the essential oils into three groups (A, B, and C) within a dissimilarity ≥ 15.0 (Figure 7). Samples of Group A were characterized by their moderate activities against all yeast strains. This group was further divided into two subgroups, A_1_ (represented by Fernana and Fondouk Djedid samples) characterized in addition by their moderate fungicidal activities against *Fusarium solani* and *Botrytis cinerea*, and A_2_ (limited to Bahra essential oil) which showed a weak fungicidal activity against mycetes strains. Group B, limited to the essential oil of plant harvested in Dhrea Ben Jouder, presented a moderate activity on mycetes strains and a very weak activity against yeasts. Group C, represented exclusively by the sample from Bou Arada, showed a good activity on *Candida glabrata*, a moderate activity against mycetes, and a poor fungicidal action on *Candida albicans* and *Candida krusei*.

**Figure 8:**
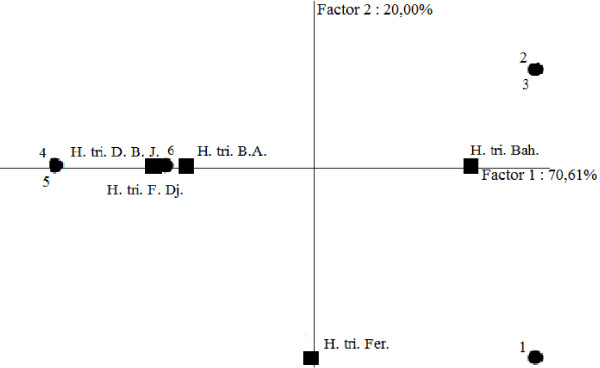
**PCA Projection of the *H. Triquetrifolium *essential oils based upon their fongicid activities against tested strains.** H. tri. B. A. *H. triquetrifolium* harvested in Bou Arada, H. tri. Bah. *H. triquetrifolium* harvested in Bahra, H. tri. D. B. J. *H. triquetrifolium* harvested in Dhrea Ben Jouder, H. tri. Fer. *H. triquetrifolium* harvested in Fernana, H. tri. F. Dj. *H. triquetrifolium* harvested in Fondouk Djedid. 1. *Aspergillus niger*. 2. *Fusarium solani*. 3. *Botrytis cinerea*. 4. *Candida krusei*. 5. *Candida albicans*. 6. *Candida glabrata*.

PCA analysis based on MFC values showed, explained 90.61% of the total variability using the first two axes. In detail, PCA horizontal axis explained 70.61% of the total variance, while the vertical axis a further 20.00% (Figure 8). While essential oils obtained from plant harvested from Bou Arada, Dhrea Ben Jouder and Fondouk Djedid negatively correlated with the first axis and with mycetes strains, they positively correlated with yeast strains. This means that they were more effective on mycetes than against yeast strains. Essential oil obtained from plant harvested from Bahra positively correlated with the first axis, but it correlated negatively with yeast strains. Sample obtained from plant harvested from Fernana negatively correlated with the second axis and positively correlated with *Aspergillus niger* (Strain 1 in Figure 8) which support its weak activity against this strain.

### Cytotoxicity test and antiviral activity

**Figure 9:**
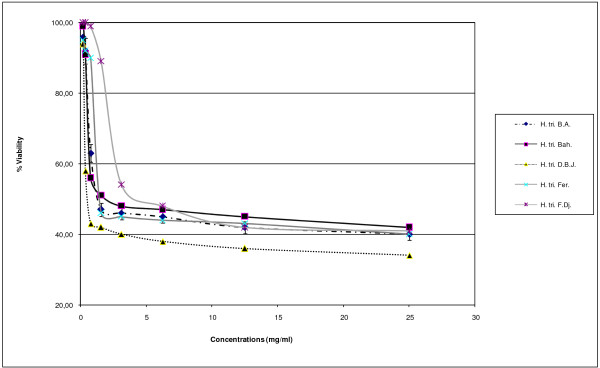
**Viability (%) of cells treated with *Hypericum *essential oils at different doses.**

The cytotoxic effect of the essential oils was dose-dependent (Figure 9). The *Hypericum* essential oils showed different cytotoxic profiles. The most cytotoxic essential oil was the one from D.B.J. (CC_50_ = 0.58 mg/mL), followed by Fer. (CC_50_ = 1.12 mg/mL), Bah. (CC_50_ = 2.50 mg/mL) and F.DJ. (CC_50_ = 4.17 mg/mL). The least cytotoxic effect was shown for the essential oil from B.A. (CC_50_ = 12.00 mg/mL) (Table 5). Unfortunately, the essential oils did not show an evident antiviral activity against coxsakievirus B3 Nancy strain, whether incubated with virus prior to infection or incubated with cells before the inoculation. We cannot exclude the antiviral activity of these essential oils against other viruses, mainly the enveloped particles, which are known to be more sensitive to environmental conditions.

**Table 5:** **50% cytotoxic doses (CC50) of *Hypericum triquetrifolium *essential oils collected from five different localities of Tunisia**

**Essential oils from different localities**	**CC50 (mg/mL) ± SD**
H. tri. B.A.	12.00 ± 0.25
H. tri. Bah.	2.50 ± 0.01
H. tri. D.B.J.	0.58 ± 0.00
H. tri. Fer.	1.12 ± 0.07
H. tri. F.Dj.	4.17 ± 0.09

H. tri. B.A.: *Hypericum triquetrifolium* collected from Bou Arada; H. tri. Bah.: *Hypericum triquetrifolium* collected from Bahra; H. tri. D.B.J.: *Hypericum triquetrifolium* collected from Dhrea Ben Jouder; H. tri. Fer.: *Hypericum triquetrifolium* collected from Fernana; H. tri. F.Dj.: *Hypericum triquetrifolium* collected from Fondouk Djedid.

## Discussion

The antibacterial activity of the essential oils of *Hypericum* species is well documented in the literature for *H. calycinum* L. [43], *H. kouytchense* H. Lév. [44], *H. coris* L. [45], *H. barbatum* Jacq.*, H. richeri* Vill. (published as *H. alpinum* WK.) [46], *H. rumeliacum* Boiss. [47], *H. hyssopifolium* ssp. *elongatum* Chaix. (syn: *H. elongatum* Ldb) [47], *H. Hyssopifolium* ssp. *hyssopifolium* Chaix. [48], *H. Hyssopifolium* ssp. *microcalycinum* Chaix., *H. Lysimachioides* Boiss var. *lysimachioides*[49], *H. Scabroides* Robson & Poulter, *H. triquetrifolium* Turra [50], *H. maculatum* Crantz [51], *H. perforatum* L. [52], *H. hirsutum* L. [53] and *H. cordatum* Vell. [54].

The antibacterial activity of *H. triquetrifolium* was previously reported only against *Bacillus brevis*, *Bacillus cereus*, *Escherichia coli PBR 322*, *Escherichia coli PUC 9*, *Pseudomonas aeruginosa* and *Staphylococcus aureus*[51]. However, the antimicrobial activity are generally influenced by the type of assay used [55].

The major components of the essential oils are found to reflect quite well their biophysical and biological features [56]. Among the main compounds detected in these essential oils, antimicrobial activities of *α*-pinene, camphene, *β*-pinene, myrcene, *ƥ*-cymene, limonene, *Ɣ*-terpinene, borneol, 1-terpinen-4-ol, *α*-terpineol, geraniol, caryophyllene oxide, longiborneol, and sclareol have been well-documented [28,57-63]. The percent values of the above compounds were 26.7, 22.8, 19.6, 19.5 and 18.5 in the *H. triquetrifolium* essential oil from B.A., F.DJ., Fer., Bah. and D.B.J., repectively [39]. These values may explain the good antimicrobial activities of the essential oils from B.A. and F.DJ.

It’s a challenge to determine which components in an essential oil are responsible on its antimicrobial activity. Although extensive research have been done within this field [63-65], an essential oil contain different identifiable components which makes it difficult to attribute this activity to one or more components without consideration of synergistic and antagonistic effects of this components. Further research is still required.

The antifungal and anticandidal activity observed in this study were higher than those obtained for antibacterial activity for all studied essential oils. The essentials oil from B.A. showed more potent fungistatic activity against candidal strains, with MIC values ranging between 0.39 *μg*/mL and 3.12 *μg*/mL, followed by the essential oils from F.DJ. and D.B.J., with MIC values ranging between 1.56 *μ*g/mL and 6.25 *μg/mL.* The essential oils from Bah. and Fer. were endowed with the least fungistatic effectiveness, with MIC values ranging between 3.12 *μ*g/mL and 12.50 *μg*/mL.

The best fungicidal effect of the essential oil from B.A. was shown against *C. glabrata* (MFC = 1.56 *μ*g/mL). The essential oil from F.DJ. had better fugicidal activity against filamentous strains (MFC = 3.12 *μ*g/mL) than the one from D.B.J. (MFC = 6.25 *μ*g/mL). The essential oils from the western localities (Bah. and Fer.) had similar fungicidal activity, with MFC values ranging between 3.12 *μ*g/mL and 12.50 *μ*g/mL. A similar study reported the antifungal activity of *H. triquetrifolium* against *Candida albicans* using disk diffusion assay [38].

Unfortunately, the tested essential oils of the Tunisian *H. triquetrifolium* did not show any clear anti-enteroviral activity. However, their activity against other viral agents cannot be excluded, as previously reported for *Hypericum connatum*, *Hypericum caprifoliatum* and *Hypericum polyanthemum* against lentiviruses [65].

## Conclusion

Antibiotic-resistant bacteria and fungi continue to be of major health concern worldwide. Bacteria have progressively developed resistance. Consequently, scientific efforts have been made to study and develop new compounds to be used beyond conventional antibiotic and antifungal therapy.

To the best of our knowledge, the present work is the first study reporting the antimicrobial activity of the essential oils of *H. triquetrifolium* from Tunisia.

These essential oils obtained from different Tunisian localities showed promising activity against bacterial and fungal strains at non-cytotoxic doses and merit worth consideration in future evaluation of Tunisian natural products for their antimicrobial potential. However, it was not possible to determine the mechanism(s) underlying these activities.

Unfortunately, these essential oils did not show any antiviral activity against coxsakievirus B3 Nancy strain, known to be resistant in the environment. However, the tested essential oils may exhibit antiviral activities against other viral strains, possibly the enveloped viruses such as herpes virus.

## Competing interests

The authors declare that there are no conflicts of interest.

## Authors’ contributions

RZ carried out the studies, acquired the data, performed the data analysis, and drafted the manuscript. KS performed the bacterial assay. YT has carried out the experimental procedures of the antifungal activities. AN performed the antiviral activity and cytotoxic test. CPL and FG helped in the analysis and interpretation of the obtained results. AM revised and supervised the work. All authors have contributed equally to this work. All authors read and approved the final manuscript.

## Pre-publication history

The pre-publication history for this paper can be accessed here:

http://www.biomedcentral.com/1472-6882/13/24/prepub
